# Study on Physical Properties of Desulfurized Electrolytic Manganese Residue Cement and Properties of Mortar

**DOI:** 10.3390/ma16114035

**Published:** 2023-05-28

**Authors:** Shichao Chen, Fang Wang, Lihua Ma, Jialing Che

**Affiliations:** 1 School of Civil and Hydraulic Engineering, Ningxia University, Yinchuan 750021, China; m17303329397@163.com (S.C.); che_jialing@nxu.edu.cn (J.C.); 2 Tian Yuan Manganese Industry Group Co., Ltd., Zhongwei 755100, China; mlharl@163.com

**Keywords:** electrolytic manganese residue, desulfurization, cement, physical properties, mechanical properties of mortar

## Abstract

The desulfurized electrolytic manganese residue (DMR) was prepared by calcination and desulfurization of industrial waste electrolytic manganese residue, and the original DMR was ground to prepare DMR fine powder (GDMR) with specific surface areas of 383 m^2^/kg, 428 m^2^/kg, and 629 m^2^/kg. The effects of particle fineness and content of GDMR (GDMR content=0%, 10%, 20%, 30%) on the physical properties of cement and the mechanical properties of mortar were studied. After that, the leachability of heavy metal ions was tested, and the hydration products of GDMR cement were analyzed using XRD and SEM. The results show that the addition of GDMR can regulate the fluidity and water requirement for the normal consistency of cement, delay the hydration process of cement, increase the initial setting and final setting time of cement, and reduce the strength of cement mortar, especially the strength of early age mortar. As the fineness of GDMR increases, the reduction of bending strength and compressive strength decreases, and the activity index increases. The content of GDMR has a significant effect on short-term strength. With the increase in GDMR content, the strength reduction degree becomes higher and the activity index decreases. When the content of GDMR was 30%, the 3D compressive strength and bending strength decreased by 33.1% and 29%. When the content of GDMR in cement is less than 20%, the maximum limit of leachable heavy metal content in cement clinker can be met.

## 1. Introduction

With the continuous development of the construction industry, the demand for cementitious materials is increasing day by day, and the annual output of cement in China in 2022 was about 2.118 billion tons [1]. Therefore, it is necessary to find other cementitious materials to meet the demand for cement production. Partial replacement of cement with solid waste is considered to be an efficient and high value-added utilization method, which can not only treat the discharged waste on a large scale but also reduce the carbon dioxide emissions from concrete production [2,3,4,5]. Patel et al. [6] studied the influence of glass powder fineness on cement properties, and the results showed that the addition of glass powder as a substitute in traditional O.P.C. cement was useful for improving various properties of the final product. At a certain water–cement ratio, 20% substitution is feasible for 63 mm glass powder, but it is limited to 10–15% for 75 mm glass powder. Zhao et al. [7] studied the dry grinding of uncontaminated marine sediments as a partial substitute for cement in mortar and concrete manufacturing, and obtained good mechanical properties. It was demonstrated that concrete C30/37 could be designed with 20% cement replaced by sediment without the use of admixture. Chen et al. [8] studied the replacement of a certain amount of cement with dolomite powder, and the results showed that the addition of dolomite powder could improve the mechanical properties and durability of glass fiber reinforced mortar. The optimal dosage of dolomite powder is 10%. Ozkan et al. [9] conducted a study on the partial replacement of cement by waste andesite dust, and the results showed that when the replacement rate was 5%, the compressive, flexural, and tensile strength of cement was similar to that before replacement.

Manganese is an important basic material in the national economy and one of the important strategic resources of the country. As a typical hydrometallurgical industry, the electrolytic manganese production industry has developed rapidly, but it has also produced serious harm to the environment. According to statistics, every ton of manganese produced will produce 8–12 tons of electrolytic manganese residue [10,11,12,13]. At present, most electrolytic manganese enterprises transport waste residue to the storage yard and build dams for wet storage. While occupying a large amount of land resources, leaching of high-concentration wastewater will cause heavy metal pollution and soil hardening. Under the condition of leaching and rainwater leaching, manganese and ammonia nitrogen in surface water will exceed the standard, which will cause great harm to the environment. In addition, the electrolytic manganese residue also contains a small amount of Cr, Cu, Ni, Pb, and As. The results show that the leaching concentrations of manganese and ammonia nitrogen reach 912.34 mg/L and 1030 mg/L, respectively, far exceeding the values specified in the reference standard [14,15,16,17]. Therefore, to reduce environmental pollution and achieve sustainable development, it is necessary to find an environment-friendly way to make full use of manganese slag.

To effectively utilize electrolytic manganese slag, many scholars have performed much in-depth research on it. Lan et al. [18] prepared a new heavy metal removal material (A-EMS) by ball milling activated electrolytic manganese slag and adding a small amount of NaOH. Du et al. [19] investigated the feasibility of using EMSW to produce steam-autoclaved bricks, and the results showed that the strength, dry shrinkage rate, frost resistance, and leaching toxicity all met the requirements. Because electrolytic manganese slag contains a large amount of gypsum and SiO_2_, some scholars use it as sulfur source, calcium source, and silicon source to prepare the cement [20]. He et al. [21] used electrolytic manganese slag and barium slag to prepare aluminosilicate cement, and its compressive strength in 3, 7, and 28 days reached 37.9, 47.3, and 60.0 MPa, respectively. Hou et al. [22] used electrolytic manganese slag to produce quasi-sulfoaluminate cementing materials. The results showed that the use of EMR for Q-SAC production is a promising way to recycle EMR because of its low firing temperature and good mechanical performance. Some scholars use electrolytic manganese slag as a raw material for brick making, geopolymers, and fertilizer. Tang et al. [23] studied the pore structure distribution of electrolytic manganese slag-based permeable brick. The internal ball and stick model was built. Wang et al. [24] prepared unsintered permeable brick with electrolytic manganese slag as the raw material, and the results showed that the 28 d splitting tensile strength and permeability coefficient reached 3.53 MPa and 3.2 × 10^−2^ cm/s. Li et al. [25] prepared EMR-FA-MK geopolymers with solid wastes such as electrolytic manganese slag (EMR), fly ash (FA), and metakaolin (MK) as raw materials for efficient fixation of heavy metals Pb^2+^ and Cd^2+^. Li et al. [26] mixed electrolytic manganese slag with Ca(OH)_2_ and activated potash feldspar to prepare autoclaved sintering-free brick, and its 1 day compressive strength reached 23.5 MPa, and the manganese ion leaching concentration was less than 0.02 mg·L^−1^. Some scholars have mixed electrolytic manganese slag with other minerals to prepare roadbed materials, and obtained good environmental protection and mechanical properties [27]. Zhang et al. [28] prepared road base materials using electrolytic manganese slag (EMR), red mud (RM), and calcium carbide slag (CS) as the main raw materials. After curing for 7 days, the compressive strength of the mixture reached 5.6 MPa.

Although a great deal of studies have been performed on electrolytic manganese slag so far, it seems that the problem of a large accumulation of electrolytic manganese slag has not been effectively solved due to poor technical feasibility, high treatment cost, or limited consumption of electrolytic manganese slag. Therefore, to eliminate the environmental pollution caused by electrolytic manganese slag at a low cost, technology is much needed to treat electrolytic manganese slag. To dispose of electrolytic manganese slag and reduce its impact on the environment, Ningxia Tianyuan Manganese Industry Co., Ltd. (Ningxia, China.). improved the process and treated the filtered acid slag produced by manganese ore with sulfuric acid in the process of producing electrolytic manganese metal, and prepared desulfurized electrolytic manganese slag after calcination and desulfurization treatments. Compared with electrolytic manganese slag, desulfurized manganese slag does not contain ammonia nitrogen, and the leaching toxicity of heavy metals and gypsum content are greatly reduced, which provides the possibility for the wide application of desulfurized manganese slag. Wang et al. [29] analyzed the feasibility of desulfurized manganese slag in detail in terms of environmental safety and economy. The research results show that it is feasible to use desulfurized manganese slag as a mineral admixture of cement from an environmental and economic point of view.

Based on this, this paper takes the desulfurized manganese slag–cement as the research object. By mixing different fineness and different content of GDMR, the effects of particle fineness and content of GDMR on the water requirement for normal consistency, fluidity, setting time, and mechanical properties of cement are compared and analyzed, and the leaching toxicity of heavy metals is detected. At the same time, XRD and SEM are used to analyze the strength mechanism of DMR cement, revealing the influence of the fineness and content of GDMR on the performance of cement.

## 2. Materials and Methods

### 2.1. Raw Materials

#### 2.1.1. Cement

P.O 42.5 Ordinary Portland Cement (OPC) and P.II 42.5 Portland Cement (PPC) are selected as the cements and their physical properties are shown in Table 1. An XRF-1800 fluorescence spectrometer (Germany BRUKER AXS Co., Ltd., Karlsruhe, Germany) was used to analyze the chemical elements in GDMR and cement, and their chemical compositions are shown in Table 2.

#### 2.1.2. Sand for Test

The sand used in the test is Chinese ISO standard sand according to GB/T 17671-1999 “Test Method for Strength of Cement Mortar (ISO Method)”.

#### 2.1.3. DMR

**Figure 1:**
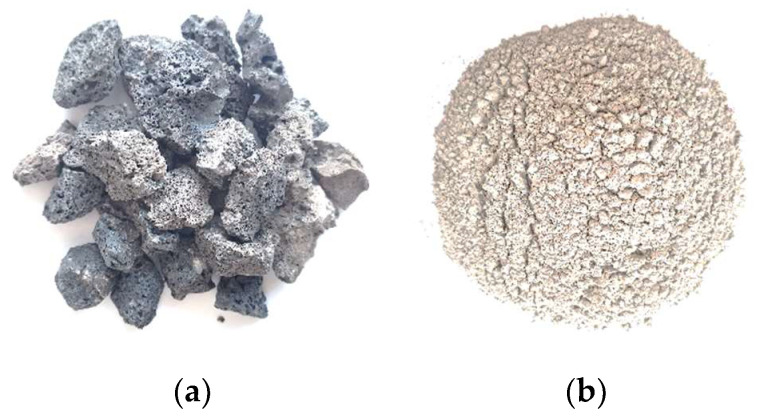
The photos of DMR. (**a**) Undisturbed DMR. (**b**) DMR after grinding.

The desulfurized electrolytic manganese residue (DMR) used in this test was provided by Ningxia Tian yuan Manganese Industry Group Co., Ltd. When sampling from the yard, the outer layer was removed by 150 mm~200 mm, and the same amount of samples was taken from more than 20 different parts, of about 20 kg, which were mixed and reduced to about 5 kg by the quartering method. The specific surface areas of the DMR samples were 383 m^2^/kg, 428 m^2^/kg, and 629 m^2^/kg; the sieve residues at 45 μm were 16.5%, 15.8%, and 2.3%, respectively. They were made by drying in a 105 °C air-blast drying oven and grinding with an SM-500 laboratory standard small mill for 50 min, 70 min, and 90 min. Figure 1 shows the photos of the original DMR and GDMR.

**Figure 2:**
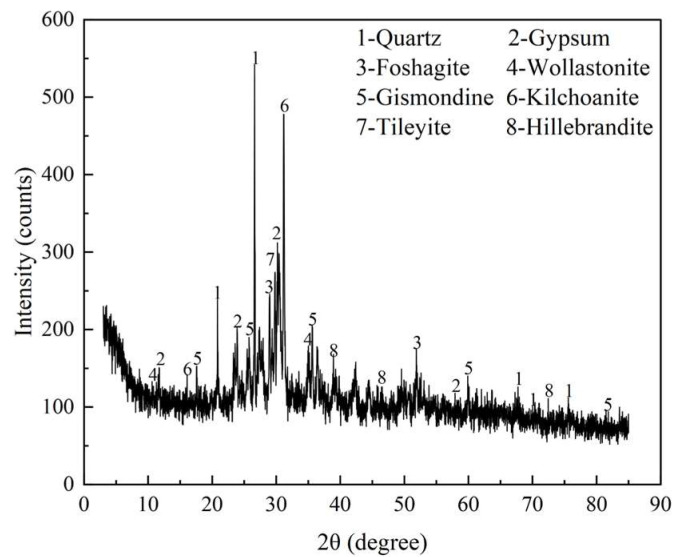
XRD patterns of DMR.

**Figure 3:**
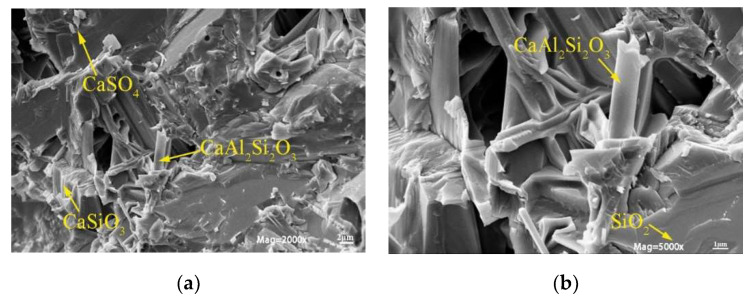
SEM patterns of DMR. (**a**) 2μm. (**b**) 1μm.

The chemical composition of DMR is shown in Table 2. The main chemical components of DMR are SiO_2_, Al_2_O_3_, Fe_2_O_3_, CaO, etc., which are similar to those of common silicate materials. They are mainly clay minerals and the content of silicon and aluminum components is high, accounting for 54% at the highest. Figure 2 is the X-ray diffraction (XRD) pattern of DMR. From the diffraction pattern of DMR, it can be concluded that there are mainly 8 different crystallized compounds, among which the diffraction peaks of SiO_2_ (PDF#00-046-1045), Ca_6_(SiO_4_)(Si_3_O_10_) (PDF#00-046-1479), and CaSO_4_·2H_2_O (PDF#97-001-5982) are sharp, indicating that they have high crystallinity and large grains. At the same time, the intensity of diffraction peaks of the quartz phase and the gypsum phase in DMR gradually weakened, which indicated that at this calcination temperature, quartz gradually became amorphous and melted with other metal elements to change into glassy silicate, while gypsum decomposed into CaO, and the main diffraction peak of low-temperature cristobalite appeared at 21°, which indicated that some quartz recrystallized into cristobalite. In order to clarify the microstructure of DMR, scanning electron microscopy was used to analyze the microstructure of DMR. Figure 3 is a scanning electron microscope observation photo of DMR at 2 μm and 1 μm scale lengths. It can be observed from Figure 3 that the microscopic morphology of DMR is mainly block, plate, plate-columnar, and fibrous aggregates at a scale length of 2 μm, and its surface is wrapped with small granular particles. They cemented to each other to form a whole, but there were some pores inside. Combining the analysis results of XRF and XRD, it can be concluded that the plate-like or plate-columnar crystals in the DMR are anorthite, the massive aggregates with the largest area in SEM photos are quartz, the dense fibrous aggregates are wollastonite, and the small dense granular particles on the surface are anhydrite.

### 2.2. Preparation of Cement Mortar

The GDMR mortar was produced by mixing different mass ratios of DMR fine powder to cement (M_cement_/M_DMR_), and the cement mortar was prepared according to 450 g cementitious material, 1350 g standard sand, and 225 g water. The cuboid specimens of 40 mm × 40 mm × 160 mm were prepared from cement mortar to test the bending strength and compressive strength. After molding and demolding, the specimen was cured at a temperature of (20 ± 1) °C and humidity of no less than 90% and then taken out after 28 days. Table 3 shows the variable design of the cement mortar mixture ratio.

### 2.3. Detection Method

According to the national standard GB/T 1346, the setting time of cement paste was measured using a Vicat apparatus (Wuxi Zhongke Building Materials Instrument Co., Ltd., Wuxi, China). When the test needle sinks to a distance of 4 mm ± 1 mm from the bottom plate, the cement reaches the initial setting state. When the test needle sinks 0.5 mm into the paste, that is, when the annular attachment cannot leave traces on the test piece, the cement reaches the final setting state. By testing the penetration of cement slurries with different water contents, the amount of water required to be added to the cement standard consistency slurry is determined, and the water requirements for normal consistency were tested in accordance with the provisions of GB/T 1346 “Standard test method for water requirement of normal consistency, setting time and soundness of the cement”.

The fluidity is determined by a cement sand fluidity tester (Shanghai Dongxing Building Material Testing Equipment Co., Ltd., Shanghai, China) (referred to as a jumping table). The fluidity shall be tested according to GB/T 2419 “Method for determination of fluidity of cement mortar”.

The cuboid specimens of 40 mm × 40 mm × 160 mm were prepared from cement mortar to test the bending strength and compressive strength. The compressive strength and bending strength of mortar specimens at different ages were measured. The loading rate of compressive strength is 2400 N/s, and the loading rate of bending strength is 50 N/s. The compressive strength and bending strength of cement mortar were tested according to GB/T 17671-2021 “Test Method for Strength of Cement Mortar”.

Cement mortar was prepared by mixing cement and GDMR fine powder (GDMR specific surface area is 383 m^2^/kg) according to M_cement_/M_DMR_. According to the limit value of heavy metal content in cement clinker leaching solution in GB/T 30760-2014, the dried test block was broken, ground using a ball mill, screened by a square hole screen, and the particles with a particle size of 0.125 mm~0.25 mm were collected as samples to be tested. According to GB/T 30810 “Determination Method of Leachable Heavy Metals in Cement Mortar”, the leachability of heavy metal ions in GDMR was determined.

The crystal phase composition of the sample was analyzed using a D8 ADVANCE high-power rotating target X-ray diffractometer (Germany BRUKER AXS Co., Ltd., Karlsruhe, Germany). When the sample reached the age to be measured, the sample was crushed and placed in ethanol to stop hydration. Then, a small amount of powder sample was prepared by grinding and sieving and dried to constant weight in a vacuum oven. The scanning parameters used in the test are as follows: the diffraction anode target is Cu target, the acceleration voltage is 40 kV, the current is 40 mA, the scanning speed is 8°/min, and the diffraction angle is 5~80°.

The microscopic morphology was observed using a Quanta 250 FEG environmental scanning electron microscope (Japan Electronics Co., Ltd., (JEOL), Tokyo, Japan.). The resolution of secondary electrons in a low vacuum and environmental vacuum mode was less than 1.4 nm, and the resolution of the energy spectrum was 127 eV.

## 3. Results and Discussion

### 3.1. Effect of GDMR on Physical Properties of Cement

#### 3.1.1. Fluidity and Normal Consistency

**Figure 4:**
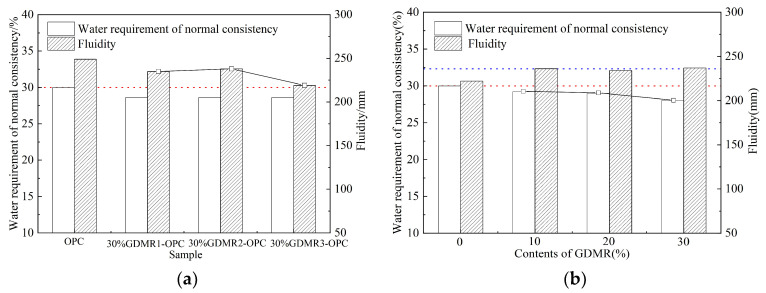
The effect of GDMR on fluidity and the water requirement for normal consistency of cement. (**a**) GDMR fineness. (**b**) GDMR content. Both red and blue dashed lines are reference lines. The red dotted line in Figure a is the water requirement of OPC at standard consistency.The red dotted line in Figure b is the water consumption of cement at standard consistency when GDMR content is 0%. The blue dashed line in Figure b shows cement fluidity when GDMR content is 10%.

**Figure 5:**
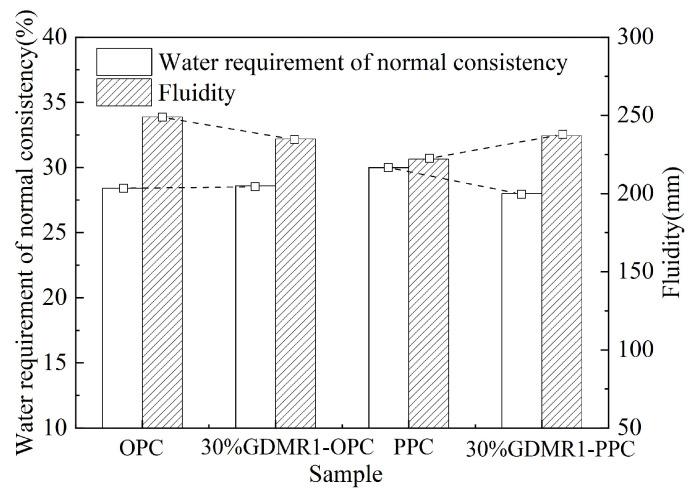
The effect of cement type on fluidity and the water requirement for normal consistency. The dashed line is a trend line, showing only the trend of change.

Water demand is an important comprehensive reference index for the application of GDMR as cement and concrete admixture in practical engineering. The water requirement of GDMR cement paste refers to the water requirement when the cement paste reaches a specific plastic state after adding water, which is expressed by the percentage of the ratio of the weight of mixing water to the weight of cement and becomes the water requirement for normal consistency of cement. The smaller the water requirement for normal consistency of GDMR cement, the higher the engineering utilization value of GDMR. The water requirement of GDMR cement mortar is usually expressed by the fluidity of standard mortar, which reflects the plasticity of cement mortar. The fluidity of mortar is related to the water requirement for normal consistency of cement but the relationship between them is not completely consistent. Figure 4 shows the influence of fineness and content of GDMR on cement fluidity and the water requirement for normal consistency, and Figure 5 shows the influence of cement types on water consumption and fluidity for normal consistency of cement mixed with GDMR. As can be seen from Figure 4 and Figure 5:

(1) GDMR fineness has little effect on the water requirement for normal consistency of cement, but has a great effect on fluidity (Figure 4a). With the increase in the specific surface area of GDMR, its fluidity first increases slightly and then decreases obviously. This is mainly due to the filling effect of GDMR in cement mortar, which releases some free water and increases its fluidity. With the further increase in the specific surface area of GDMR, the released free water is absorbed by the finer GDMR particles, which ultimately leads to the decrease in mobility [30].
(2) As the content of GDMR increases gradually, the water requirement for normal consistency of cement does not change, showing a slow downward trend, and having little influence on fluidity (Figure 4b).
(3) The influence of the GDMR on water requirement for normal consistency and fluidity of OPC and PPC cement is just the opposite. For PPC, GDMR can improve its fluidity and reduce its water requirement for normal consistency. However, for OPC, GDMR will reduce its fluidity and increase its water requirement for normal consistency. The fluidity and water requirement for normal consistency of two kinds of cement mixed with GDMR are basically the same, which shows that GDMR can regulate the fluidity and the water requirement for normal consistency of cement.

#### 3.1.2. Setting Time

Table 4 shows the influence of fineness and content of GDMR on cement setting time. The setting time ratio is the ratio of the setting time of desulfurized manganese slag cement paste under standard consistency to that of ordinary Portland cement paste, which can be used to describe the setting process of cement mixed with GDMR. As can be seen in Table 4:

(1) With the increase in GDMR specific surface area, the initial setting time and final setting time of cement are prolonged gradually. Compared with the final setting time, GDMR fineness has a more significant influence on the initial setting time of cement. Moreover, with the increase in GDMR fineness, the trend of the increasing cement setting time ratio decreases.
(2) With the increase in GDMR content, the initial setting time and final setting time of cement are prolonged gradually, and the setting time ratio is also increased gradually. When the content of DMR is more than 20%, the setting time of cement is prolonged significantly. Compared with the final setting time, the influence of GDMR content on the initial setting time of cement is more significant.
(3) The GDMR can prolong the setting time of the two kinds of cement significantly. Compared with the final setting time, the influence of GDMR on the initial setting time of the two kinds of cement is more significant.

As GDMR is a weak pozzolanic material, the content of cement in the cementing material will be reduced when it is partially replaced by cement, while the reaction of the volcanic ash material generally occurs in the middle and late hydration period. Therefore, the setting time of the paste mixed with GDMR is longer than that of the pure cement.

### 3.2. Effect of GDMR on Mechanical Properties of Cement Mortar

#### 3.2.1. Strength Activity Index

(1) Fineness and strength activity index

**Figure 6:**
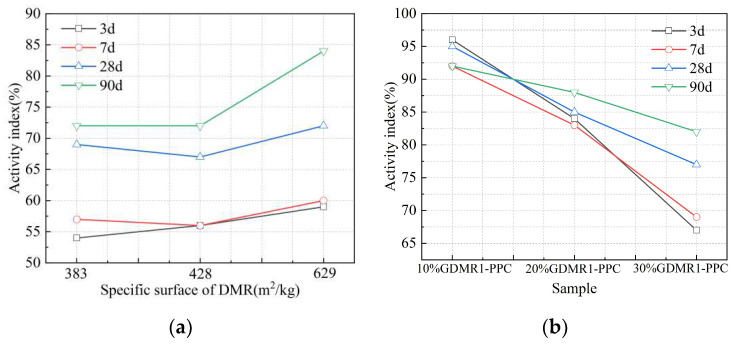
The effect of GDMR on the strength activity index. (**a**) GDMR fineness. (**b**) GDMR content.

Siliceous or aluminosiliceous materials have no or only weak cementitious properties, but they will combine with CaO to form hydraulic solids in the presence of water, which is called the pozzolanic property. GDMR is a kind of inactive admixture with weak pozzolanic properties, so the pozzolanic property can be characterized by the strength activity index. The strength activity index is the ratio of the compressive strength of test mortar to the compressive strength of contrast mortar. It can be analyzed from Figure 6a that there are differences in the strength activity index of GDMR samples with different fineness. With the extension of grinding time, the specific surface area of GDMR increases gradually, the sieve residue at 45 μm decreases obviously, and the strength activity index of GDMR cement increases slowly at different ages. Compared with the strength activity index of 3 d, 7 d, and 28 d, the strength activity index of 90 d of GDMR increased significantly, and the specific surface area and sieve residue of 45 μm had a good correlation with the strength activity index of GDMR cement. At the same time, comparing the strength activity index of OPC and GDMR cement (30%), the strength activity index of GDMR cement at 3 d, 7 d, 28 d, and 90 d is lower than that of ordinary Portland cement by 46%, 43%, 31%, and 28%, respectively. Under the condition that the specific surface area of powder is basically the same, this proves that the addition of GDMR will significantly reduce the strength activity index of cement, especially affecting the strength activity index at an early age.

(2) Content and Strength Activity Index

The test results of the strength activity index of GDMR cement mortar with different dosages (0%~30%) are shown in Figure 6b. From the test results, it can be concluded that for the same fineness of GDMR, with the increase in the content of GDMR, the strength activity index of cement mortar gradually decreases. When the content of GDMR is more than 10%, the strength activity index of 3 days, 7 days, and 28 days is less than 90%. Compared with the long age (90 d) strength activity index, the content of GDMR has a more significant influence on the early age (3 d, 7 d) strength activity index. By comparing the strength activity index of GDMR cement with Portland cement, it can be concluded that when the content of GDMR in Portland cement is less than 10%, the strength activity index of early age (3 d, 7 d) GDMR cement is basically the same as that of Portland cement, and the strength activity index at 28 d and 90 d is about 8% different from that of Portland cement. When the content of GDMR is more than 10%, the strength activity index of cement mortar decreases significantly. A comprehensive comparison of the effect of the content of GDMR on the activity index of cement shows that when the content of GDMR is less than 10%, the strength activity index of early age GDMR cement is not much different from that of Portland cement, and the strength activity index at 28 d and 90 d is roughly 93% and 91% of that of PPC, respectively, while when the content of GDMR is more than 10%, the strength activity index of cement at different ages decreases obviously.

#### 3.2.2. Compressive Strength

**Figure 7:**
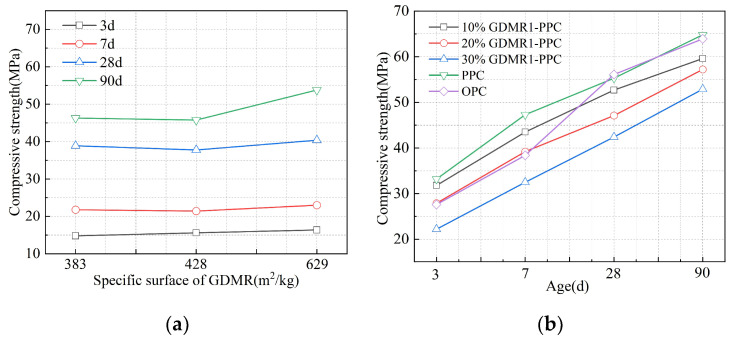
The effect of GDMR on the compressive strength of mortar. (**a**) GDMR fineness. (**b**) GDMR content.

**Figure 8:**
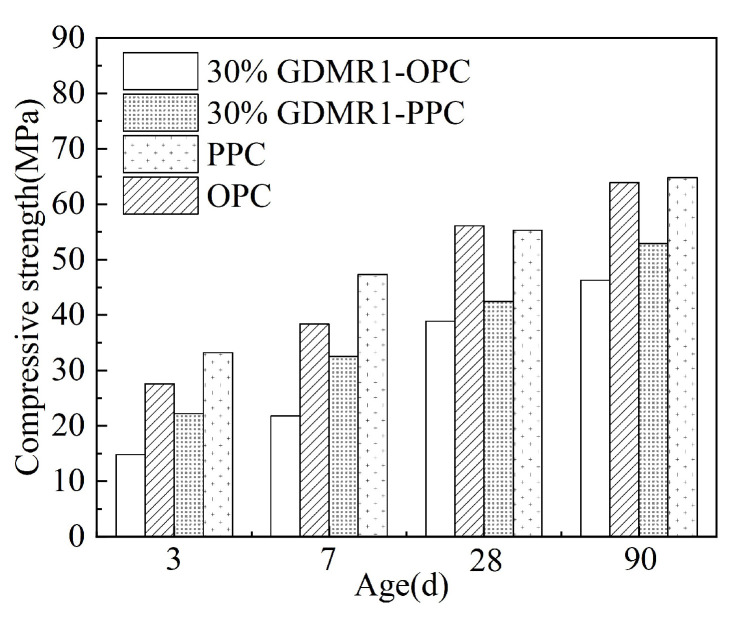
Influence of cement types on compressive strength of mortar.

Figure 7 and Figure 8 show the influence of fineness and content of GDMR on the compressive strength of cement and the influence of cement types on the compressive strength of cement mixed with GDMR.

(1) As can be seen from Figure 7a, with the gradual increase in fineness of GDMR, the compressive strength of cement mortar at all ages shows an upward trend and has a significant influence on the compressive strength at later ages. With the increase in specific surface area of desulfurized manganese slag, it will have a dense filling effect and increase its activity at the same time. Therefore, with the gradual increase in fineness of desulfurized manganese slag, its mechanical properties will be improved to a certain extent. Compared with the compressive strength, the fineness of GDMR has little influence on the bending strength of cement mortar. Comparing the mortar strength of OPC with that of GDMR cement, the compressive strength of GDMR cement at 3d, 7 d, 28 d, and 90 d is lower than that of OPC at 12.8 MPa, 16.5 Mpa, 17.2 Mpa, and 17.6 MPa, respectively. Under the condition that the specific surface area of powder is basically the same, this proves that the addition of GDMR will significantly reduce the mortar strength of cement, especially the mortar strength at an early age.
(2) As can be seen from Figure 7b, with the increase in the content of GDMR, the compressive strength of cement mortar decreased by 4.2%, 16%, and 33.1% at 3 d; 8%, 17.1%, and 31.3% at 7 d; 4.7%, 14.8%, and 23.3% at 28 d; and 8%, 11.7%, and 18.4% in 90 d, respectively. It can be seen that the increase in dosage has a significant effect on the compressive strength of cement mortar at an early age (3 d, 7 d). When the content of GDMR is more than 10%, the compressive strength of cement mortar decreases obviously. Comparing the compressive strength of OPC and GDMR cement at different ages, the compressive strength of cement mortar at an early age (3 d, 7 d) is basically the same as that of OPC when the content of GDMR is 20%. When the content of GDMR is less than 20%, the compressive strength of cement mortar at early age (3 d, 7 d) is slightly higher than that of OPC, while the strength of 28 d and 90 d mortar is lower than that of OPC, with the difference of 7% and 8.6%, respectively. Due to the increasing content of GDMR, the amount of cement decreases, the hydration products gradually decrease, the Ca(OH)_2_ generated by cement hydration decreases, and the excitation effect on GDMR is relatively weakened, resulting in a decrease in strength [31].
(3) As can be seen from Figure 8, the influence of GDMR on the compressive strength of OPC mortar at various ages is much higher than that of PPC.

#### 3.2.3. Bending Strength

**Figure 9:**
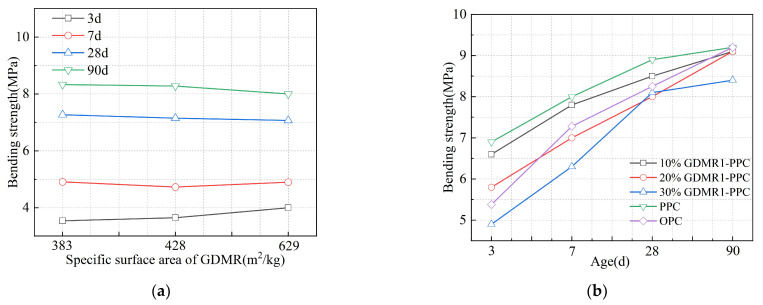
The effect of GDMR on the bending strength of mortar. (**a**) GDMR fineness. (**b**) GDMR content.

**Figure 10:**
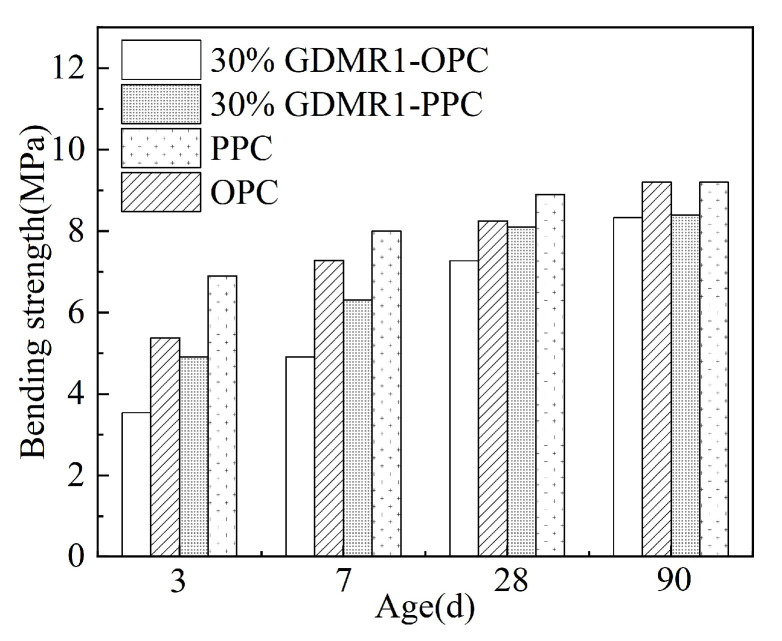
Effect of cement types on bending strength of mortar.

Figure 9 and Figure 10 show the influence of the fineness and content of GDMR on the bending strength of cement mortar and the influence of cement types on the bending strength of cement mixed with GDMR.

(1) Comparing the bending strength of cement mortar at different ages, it can be seen that the bending strength of cement mortar at younger age increases with the increasing fineness of GDMR, while the bending strength at later ages decreases.
(2) The bending strength of cement mortar decreased by 4.3%, 16%, and 29% at 3 days; 2.5%, 12.5%, and 21.3% at 7 days; 4.5%, 10.1%, and 9% at 28 days; and 1.1%, 1.1%, and 8.7% at 90 days with the increase in the content of DMR. It can be seen that the content of GDMR has a similar influence on bending strength and compressive strength, and both have a greater influence on early-age strength. When the content of GDMR is less than 20%, its 90-day bending strength is basically the same as that of OPC and PPC.
(3) The effect of GDMR on the bending strength of PPC at different ages is almost the same.

### 3.3. Heavy Metal Ion Leachability

Table 5 shows the leachability of heavy metal ions in cement mortar with different dosages of GDMR (0~30%). It can be seen from Table 5 that when the content of GDMR is 0~20%, the heavy metal content in the leaching solution of GDMR cement mortar is lower than the limit value of heavy metal content in the clinker leaching solution. However, with the increasing content of GDMR, there is a danger that manganese dissolution in GDMR cement exceeds the standard. The contents of leachable heavy metals such as arsenic, lead, cadmium, and zinc in GDMR cement are basically the same as those in PPC, while the leachable contents of chromium, copper, nickel, and manganese are obviously higher than those in PPC. The radioactivity of desulfurized manganese slag cement meets the technical requirements of GB6566 “Radionuclide Limits of Building Materials”.

Therefore, when the content of GDMR in PPC is less than 20%, the content of leachable heavy metals in cement meets the limit requirements of relevant national standards, but it is still necessary to pay attention to the impact of chromium, copper, nickel, and manganese dissolution on the environment.

### 3.4. Microscopic Analysis of GDMR Cement Paste

**Figure 11:**
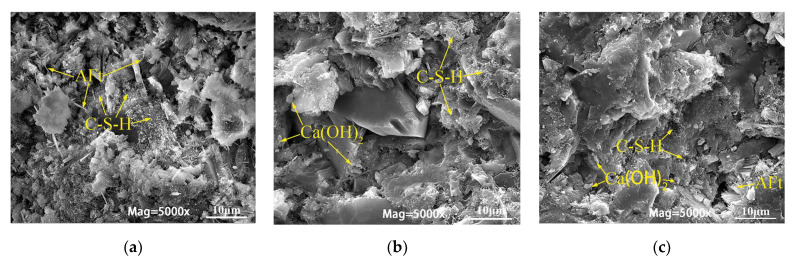
SEM analysis results of hydration products of GDMR cement paste at 1 d, 3 d, and 7 d ages. (**a**) 1 d. (**b**) 3 d. (**c**) 7 d.

**Figure 12:**
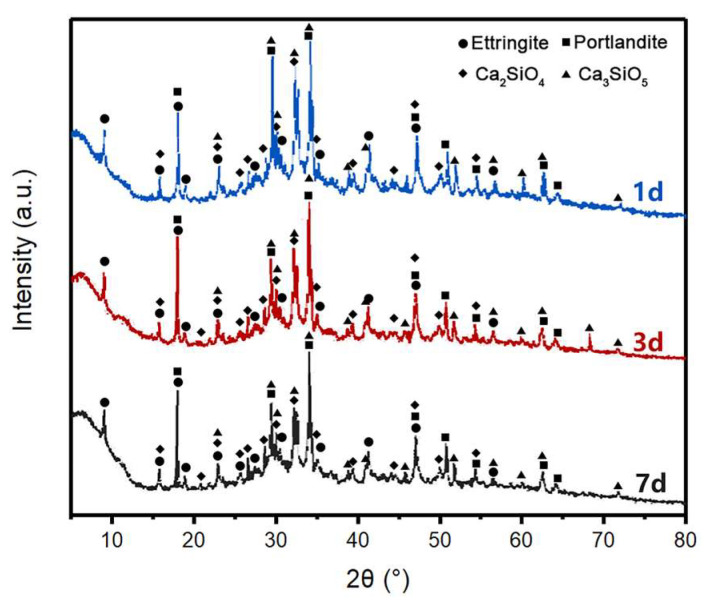
XRD analysis results of hydration products of GDMR cement paste at 1 d, 3 d, and 7 d ages.

SEM and XRD analysis results of hydration products of GDMR cement paste at 1 d, 3 d, and 7 d ages are shown in Figure 11 and Figure 12.

It can be seen from Figure 11a that more needle-columnar ettringite is formed in the cementitious system, and ettringite is connected to form a skeleton. At the same time, a small amount of C-S-H gel is also formed, but the hydration structure is still relatively loose. After hydration for 3 days, the formation of C-S-H gel and calcium hydroxide in the cementitious system gradually increased, and gradually filled in the voids, which made the structure of the cement slurry become dense. However, it is obvious that the interface between hydration products and GDMR is very obvious, which indicates that GDMR has low activity, which finally makes its strength lower than that of reference sample (Figure 11b). With the development of the reaction, the ettringite grew stronger after 7 days of hydration, and the amount of C-S-H gel and calcium hydroxide in the cementitious system increased. The unreacted GDMR was encapsulated by hydration products, and its structure became denser.

As can be seen from Figure 12, the hydration products of cement mixed with GDMR are mainly ettringite (PDF#00-041-1451), calcium hydroxide (PDF#00-004-0733), and hydrated calcium silicate. Among them, ettringite and calcium hydroxide have strong diffraction peaks. When hydrated for 1 d, the diffraction peaks of ettringite and calcium hydroxide are weak, and there is much unhydrated tricalcium silicate (PDF#00-016-0406) and dicalcium silicate (PDF#00-001-1029) in the cementitious system, which leads to reducing the early strength of cement. When hydrated for 3 and 7 days, the diffraction peaks of ettringite increased obviously, the content of tricalcium silicate and dicalcium silicate decreased, the hydration speed increased, and the macroscopic properties showed that the cement strength increased gradually. Due to the weak pozzolanic activity of GDMR, the pozzolanic reaction of GDMR will consume a certain amount of Ca(OH)_2_ to produce calcium silicate hydrate (C-S-H) gel over time, which leads to the reduction in Ca(OH)_2_ content. Compared with curing for 3 d, the intensity of tricalcium silicate and Ca(OH)_2_ diffraction peaks decreased slightly at a curing age of 7 days, indicating that there was more hydration of cement clinker at this time. The pozzolanic activity of GDMR mainly occurs in the later stage of hydration. It can be seen that the dilution of GDMR reduces the content of cement clinker in the composite cementitious material, thus delaying the early hydration process of the composite cementitious material [32]. Therefore, the addition of GDMR will delay the hydration process of cement, and then affect the strength of cement mortar at an early age. This is consistent with the change law of compressive strength with the dosage of GDMR.

## 4. Conclusions

(1) GDMR can adjust the fluidity of cement and the water requirement for normal consistency. When using GDMR–cement to prepare concrete, the regulation of mineral admixture GDMR and superplasticizer on concrete workability should be fully considered in the design of concrete mix proportion. The unit water consumption and water–cement ratio should be determined according to compressive strength, and appropriate modifications should be made to give consideration to durability.
(2) When the content of GDMR in cement is less than 20%, the leachable heavy metal content in cement is lower than the maximum limit value of leachable heavy metal content in clinker, but attention should be paid to the environmental impact of chromium, copper, nickel, and manganese dissolution.
(3) With the increase in the specific surface area of GDMR, the initial setting time and final setting time of cement are extended by 12 min and 15 min, respectively. The cement set time ratio increased by 0.06.
(4) The addition of GDMR will delay the hydration process of cement, thus reducing the strength of cement mortar, especially at early ages. When the dosage is 20%, the compressive strength of 3 d and 7 d cement decreases by 16% and 17.1%, respectively. The greater the fineness of GDMR, the smaller the reduction degree of flexural strength and compressive strength, and the higher the activity index. However, the fineness should not be too large, when it is too fine it will increase the production cost and will have an adverse effect on the cement fluidity. At the same time, the content of desulfurization manganese slag should be controlled within 20%.

## Figures and Tables

**Table 1:** Physical properties of cement.

Sample	45 μm Fineness (%)	Standard Consistency (%)	Initial Setting Time (min)	Final Setting Time (min)	Bending Strength (MPa)		Compressive Strength (MPa)	
					3d	28 d	3d	28 d
OPC	0.7	28.4	165	260	5.4	8.3	27.6	56.1
PPC	0.6	30	142	202	6.9	8.9	33.2	55.3

**Table 2:** Chemical composition of cement and DMR.

Sample	SiO_2_	CaO	Al_2_O_3_	MnO	Fe_2_O_3_	MgO	SO_3_	Na_2_O	K_2_O
OPC	22.35	59.22	6.49	-	2.73	4.01	2.22	-	-
PPC	22.06	59.76	6.25	-	2.81	3.98	2.31	-	-
DMR	40.22	31.14	7.98	6.63	6.41	1.89	1.36	1.31	1.25

**Table 3:** Variable design of mix proportions of GDMR cement.

Sample	Cement	M_cement_/M_DMR_	DMR Specific Surface Area (m^2^/kg)	45 μm Sieve Allowance (%)	80 μm Sieve Allowance (%)
PPC	PPC	100:0	/	/	/
30%GDMR1-OPC	OPC	70:30	383	16.5	4.2
30%GDMR2-OPC	OPC	70:30	428	15.8	3.6
30%GDMR3-OPC	OPC	70:30	629	2.3	1.6
OPC	OPC	100:0	/	/	/
10%GDMR1-PPC	PPC	90:10	383	16.5	4.2
20%GDMR1-PPC	PPC	80:20	383	16.5	4.2
30%GDMR1-PPC	PPC	70:30	383	16.5	4.2

**Table 4:** Influence of GDMR fineness and dosage on cement setting time.

Sample	Specific Surface Area of GDMR (m^2^/kg)	Initial Setting Time (min)	Final Setting Time (min)	Initial Setting Time Ratio	Final Setting Time Ratio
PPC	-	165	260	1.00	1.00
30%GDMR1-OPC	383	220	305	1.33	1.17
30%GDMR2-OPC	428	228	314	1.38	1.21
30%GDMR3-OPC	629	232	320	1.40	1.23
OPC	-	142	202	1.00	1.00
10%GDMR1-PPC	383	162	217	1.14	1.07
20%GDMR1-PPC	383	175	230	1.23	1.14
30%GDMR1-PPC	383	185	255	1.30	1.26

**Table 5:** Heavy metal ion leachability of GDMR cement mortar mg/L.

Sample	Arsenic (As)	Lead (Pb)	Cadmium (Cd)	Chromium (Cr)	Copper (Cu)	Nickel (Ni)	Zinc (Zn)	Manganese (Mn)
PPC	0.0004	<0.001	<0.001	0.009	0.005	0.015	0.127	0.46
10%GDMR1-PPC	0.0010	0.003	<0.001	0.044	0.050	0.116	0.167	0.86
20%GDMR1-PPC	0.0010	0.002	<0.001	0.030	0.034	0.122	0.148	1.00
30%GDMR1-PPC	0.0009	0.001	<0.001	0.015	0.017	0.127	0.128	1.12
Limits	0.1	0.3	0.03	0.2	1	0.2	1	1

## Data Availability

The data presented in this study are available on request from the corresponding author.
